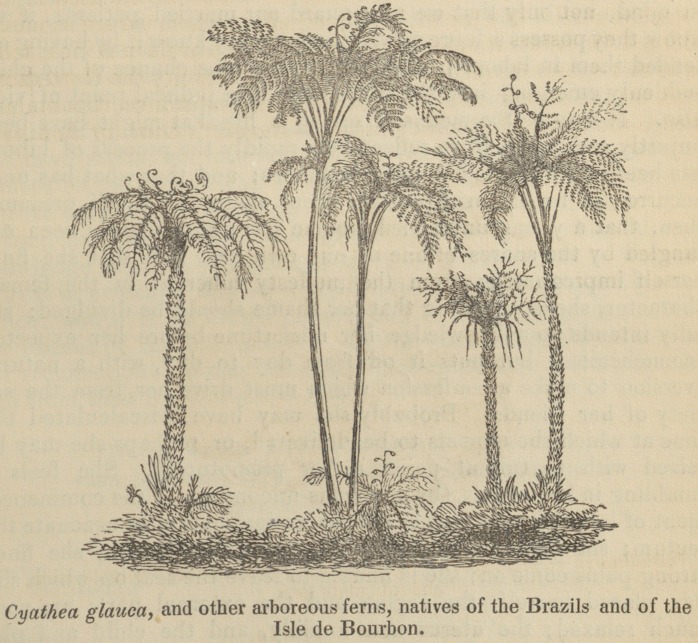# Collectanea: Miscellaneous

**Published:** 1834-01-01

**Authors:** 


### MISCELLANEOUS.

### STATISTICS OF EUROPEAN MORTALITY.

M. Moreau-de-Jonnés lately read a memoir on this subject at the Royal Academy of Sciences. The causes which act on the population of Europe, says the author, have a much greater influence on mortality than on reproduction. The maximum of births is barely double the minimum; but, in deaths, the former is almost treble the latter (22, 59,) in ordinary times. The annual mortality in the Roman states, and the former possessions of Venice, is 1 in 30. In Italy, Greece, and Turkey, 1 in 30. In the Netherlands, France and Prussia, 1 in 39. In Switzerland, the Austrian Empire, Portugal, and Spain, 1 in 40. In Russia, in Europe, and Poland, 1 in 44. In Germany, Denmark, and Sweden, 1 in 45. In Norway, 1 in 48. In Ireland, 1 in 53. In England 1 in 58. In Scotland and Ireland, 1 in 59.

It may be remarked, that there are two principal causes which determine the rate of mortality; these are climate and civilization. A cold, and even a rigorous climate, is favorable to long life, and so is the sea-coast with a low temperature. The regions of the South, though their climate seems favourable to the human species, are quite the reverse. Those parts of the torrid zone whose mortality has been calculated, show the destructive tendency of a high temperature. Thus, at Batavia, in lat. 6° 10′, the annual mortality is 1 in 26½. In Trinidad, lat. 10° 10′, 1 in 27. In St. Lucia, lat. 13° 54′, 1 in 27. In Martinique, lat. 14° 44′, 1 in 28. In Guadaloupe, lat. 15° 69′, 1 in 27. In Bombay, lat. 18° 86′, 1 in 20. At the Havannah, lat. 23° 11′, 1 in 33.

The resisting power of life differs between the tropics according to the races of men, and its duration at the same place in one race is double or triple of what it is in another. Thus, at Batavia, in 1805, the Europeans lost 1 in 11; the slaves, 1 in 13; the Chinese, 1 in 29; the Javanese, 1 in 40. At Guadaloupe, from 1816 to 1824, the whites lost 1 in 23½; the freedmen, 1 in 35. At Martinique, in 1817, the whites lost 1 in 24; the freedmen, 1 in 33. We may compare with this the annual mortality in Madeira, the only tropical establishment of the temperate zone. Heberden calculated it at 1 in 50.

The greater or less perfection of the social economy exercises as powerful an influence on the mortality as climate does. Thus, in Sweden, the annual mortality, from 1754 to 1763, was 1 in 34; but, from 1820 to 1825, was only 1 in 45. A great decrease may also be remarked in Denmark, Germany, Russia, Holland, and Italy. In Great Britain, from 1787 to 1789, the mortality was 1 in 43; but, from 1800 to 1804, only 1 in 47. In England alone, in 1690, it was 1 in 53; in 1821, 1 in 58. In France, in 1776, it was 1 in 25½; in 1825-1827, 1 in 39½. The mortality has been stationary for thirty years in Russia and Norway, and has increased in the kingdom of Naples. Eighty years ago, Greesmilch calculated the average mortality of the whole of Europe at 1 in 36. According to M. Moreau, it is only 1 in 40 at the present day, so that it would have diminished one-ninth. But the author of the memoir thinks that Greesmilch very much underrated the mortality at the time he wrote, so that the diminution has in fact been much greater. —*Archives Génerales, Septembre.*

This is an interesting paper, but it appears to have been imperfectly reported. Thus, in stating that the maximum of deaths in Europe is nearly treble the minimum, 1 in 22 and 1 in 59 are given as the extremes; but, in the detailed account, no European state appears with a mortality of 1 in 22; the mortality of Ireland is first given alone, and then joined with that of Scotland; and the following sentence is defective: "La résistance de la vie diffère entre les tropiques, selon les races d'homme, et sa durée est dans le même lieu double ou triple de ce qu'elle est pour les autres." The words *pour une race* should be inserted after *triple*: in translating we have supplied the omission.—*Edit. Med. Quart. Review.*

### ST. VITUS'S DANCE.

St. Vitus's dance was so called, because those who were affected with this disease used to go in pilgrimage to St. Vitus, the patron saint of the convent of Korbey, in order to request his aid.—*Jörg's Kinderkrankheiten,* p. 837.

### PROMETHEUS A DISTILLER.

I shall conclude this section on the diseases of the liver, induced by spirituous liquors, with the well-known story of Prometheus, which seems indeed to have been invented by physicians in those ancient times when all things were clothed in hieroglyphic, or in fable. Prometheus was painted as stealing fire from heaven, which might well represent the inflammable spirit produced by fermentation, which may be said to animate or enliven the man of clay; whence the conquests of Bacchus, as well as the temporary mirth and noise of his devotees. But the after punishment of those who steal this accursed fire, is a vulture gnawing the liver; and well allegorizes the poor inebriate, lingering for years under painful hepatic diseases.—*Darwin's Zoonomia,* Vol. I. p. 356.

### *Letter from* DR. SOMERVILLE *to* DR. HASTINGS, *on the Operation of the Anatomy Act.*

*5, Saville row; 2d July,* 1833.

My dear sir: I had anticipated, with much satisfaction, the prospect of being present at the ensuing meeting of the Medical Association at Bristol; not only from the opportunity thus afforded me of meeting you, and many of my old friends, but also in expectation that the bill which has been entrusted by government to my care might have received greater support, and be made more generally useful to my professional brethren, if its operation hitherto, as well as its provisions, were more generally explained.

Disappointed in the expectation, I have not hesitated to call your attention to this subject, knowing how much you have at all times at heart the advancement of your profession, and the education of those who are about to enter upon it.

The Anatomy Bill can scarcely be said to have been in operation from October last; for, having been recently passed, before the commencement of the winter courses, the teachers, as well as the public, had to enter on a discussion about the propriety of getting a supply from the sources contemplated by the bill; yet it is a fact, equally agreeable as it is surprising, that, notwithstanding the natural abhorrence of the public at the bare thought of dissection, a bill, essentially depending for its efficiency upon public feeling, should have met with such decided success. In London alone upwards of five hundred bodies have, during the last season, been supplied to the anatomical schools from parochial institutions.

To me it is more particularly gratifying to be enabled to add, that no untoward circumstance has yet occurred to give a moment's uneasiness to me: this is the more particularly gratifying, as it serves to shew that the system of management, while it affords the utmost protection to the public, has nothing in it to shock the feelings of the poor, while at the same time it is an effectual check to improper proceedings. This success I am led to attribute to the following circumstances:

1st. Carrying with us the feelings of the overseers, and of parish authorities, by shewing them the necessity of protecting the study of anatomy.

2dly. The avoidance of every circumstance calculated to give a shock to the feelings of the poor; and for this reason the bodies have always been removed by undertakers, in coffins, as if for the purpose of interment; and, on the burial of the remains, the utmost precaution has been taken as to the observance of the usual rites, with this difference only, that the appearances are made more respectable than those of paupers. Many of the more respectable inmates of workhouses, seeing the decency observed in these transactions, have voluntarily given up their bodies; and the relatives of others, grateful to their parochial surgeons, have asked to have their bodies sent to the schools for partial examination, as it is termed, when the teacher is requested not to disfigure the features, and to return the body within fourteen days.

It is impossible for me to conclude this description, without acknowledging, with pride, the unremitting zeal and anxious efforts of the home secretary to promote, by every means in his power, the operation of the bill, so as to make it of the utmost service to the profession. As to the provincial schools, there are difficulties which make me anxiously request the sense of the meeting, in regard to any suggestions which may appear to them calculated to remove them.

The obstacles in obtaining a supply in small towns are obvious; for, not only are the guardians of the poor reluctant to incur the odium of assisting dissection, but the actual number of unclaimed bodies is necessarily very small. To this circumstance I am bound to attribute the want of success which has attended the school at Exeter, which I the more sincerely regret, because, during the many years I was attached to the school of anatomy in Windmill street, I had the most convincing proofs of the proficiency of the pupils from that school.

The act is so framed as to prevent the removal of bodies from one town to another: indeed, the risks which attend such removals so far overbalance any advantage, that, for the sake of the community, as well as of the large schools, I believe such a permission to be highly inexpedient. It is to these difficulties to which I beg more particularly to call your attention.

It has often been suggested that the act might be made compulsory; but the objections to this are very strong, as it would not only very materially increase the prejudice against dissection, but it would be assuming a power quite foreign to every liberal feeling; and I do not know on whom the government could rely to carry such an act into effect. As to almost all the other provincial schools, the success has been of such a nature as to give the most encouraging hope that, by a continuance of the good understanding between the teachers and local authorities, this most essential part of medical education will no longer be made to depend upon the violation of the grave, or the caprices of resurrection-men.

So fully has the government been impressed with the belief that anatomy could not be prosecuted with safety until the practice of exhumation was put a stop to, that the most peremptory orders have been given for this purpose; and to the successful prosecution of several individuals engaged in this traffic we owe much of the success we have attained.

In conclusion, I have only to offer my humble efforts in rendering useful the enlightened and liberal measure of Mr. Warburton, who, in every transaction in which the advancement of our profession has been concerned, has ever been the foremost to give his utmost assistance.

Believe me, my dear sir, with great esteem, yours, most faithfully, JAMES C. SOMERVILLE.—*Medical Gazette.*

[We hear that there has been a great deficiency of subjects this season.—EDIT.]

*Phœnicia* formerly produced the best dates known, the date-palm was hence called *Phœnix.* It grows abundantly in Egypt, Arabia, Persia, and the neighbouring countries, and contributes largely to the support of the inhabitants, being in many places, as in Upper Egypt, the chief source of food.

The Date-palms being diœcious, (*i. e.* the stamens and pistils being not only in separate flowers, but growing on different trees,) the crops entirely fail, or the fruit is degenerate and unfit for food, if unseasonable weather, or any accident, should prevent the pollen of the stamineous plants having access to the flowers of the fruit-bearing ones. To ensure the fertilization of the seeds the Arabs have long been accustomed to gather the stamineous clusters and hang them over the pistilliferous flowers, and even to lay up stores of pollen from year to year. At the season when this is done a feast is held, called the Marriage of the Palms, of which Haselquist has given a very interesting account; and it is stated, that so well do the half-savage tribes know the importance of this process, that, during inroads into hostile countries, they cut down the stamen-

*Phœnix dactylifera.*

bearing palms, as one of the most severe injuries tney can inflict. Desfontaines was witness to such an act of vengeance, and Kœmpfer relates that the threat of so doing once warded off an invasion; for, after describing the artificial fecundation of the date, he adds:

"I remember it happened in my time that the Grand Signior meditated an invasion of the city and territory of Bassora, which the prince of the country prevented, by giving out that he would destroy all the male palm-trees on the first approach of the enemy, and by that means cut off from them all supplies of food during the siege."

The extensive importance of the date-tree is, says Dr. Clarke, one of the most curious subjects to which a traveller can direct his attention. A considerable part of the inhabitants of Egypt, Arabia, and Persia, subsist almost entirely on its fruit; as a luxury they make a conserve of it, and they boast also of its medicinal virtues, esteeming it a tonic. Upon the abortive fruit and upon the ground date-stones, the camels are fed. From the leaves they make couches, baskets, bags, mats, brushes, and fly-flaps; from the trunk cages for their poultry, and fences for their gardens, and other parts of the tree furnish fuel. From the fibrous webs at the bases of the leaves, thread is procured which is twisted into ropes and rigging, and from the sap, which is collected by cutting off the head of the palm and scooping out a hollow in its stem, a spirituous liquor is prepared. Three or four quarts of sap may be obtained daily from a single palm for ten days or a fortnight, after which the quantity lessens, until, at the end of six weeks or two months, the stem is exhausted. So numerous being the uses of this palm, it is no wonder that it is highly prized, or that the native literati should have celebrated in verse and prose (as Gibbon informs us,) the 360 uses to which the trunk, the stalks, the leaves, the juice, and the fruit, have been skilfully applied.—*Ibid.*

### ORDER OF THE ERUPTION OF THE FIRST TEETH.

Dr. Ashburner supposes that the following is the order in which the first teeth most frequently appear:

Two central lower incisors,
Two central upper incisors,
Two lateral lower incisors,
Two lateral upper incisors,
Four first molar teeth,
Two lower canine teeth,
Two upper canine teeth,
Last four molar teeth.

A correspondent of the "Medical Gazette," however, gives a table drawn up by Sir Richard Croft, which he thinks more accurate: *judicent peritiores.*

| Molars. | | Canine. | Incisors. | | | | Canine. | Molars. | | |
|---|---|---|---|---|---|---|---|---|---|---|
| 9 | 5 | 7 | 3 | 2 | 2 | 3 | 7 | 5 | 9 | Upper jaw. |
| 10 | 6 | 8 | 4 | 1 | 1 | 4 | 8 | 6 | 10 | Under jaw. |

### ENGLISH EYE-SURGERY; A SKETCH. BY PROFESSOR WALTHER, OF MUNICH.

The first, and, in the common estimation, the best oculist now alive in London, is Mr. H. Alexander. He was formerly the pupil, and for many years the assistant, of Phipps, who, after having practised, as an oculist, for a long space of time with success and celebrity, retired while yet a hale and hearty man, was raised to the dignity of a baronet, married an extremely rich lady, drew up entirely with the nobility, and left as a legacy to his assistant a most capital practice.

Even the external appearance and the whole demeanour of Alexander are significant of sudden elevation from an inferior station, without scientific instruction. Medicine and surgery he appears never to have studied. With the lessons of his master, merely, has he become a bustling, clever oculist. As such he not only commands a very extensive private practice, embracing as widely the genteel part of the community as the middle ranks, but he has also the care, almost exclusively, of the most important and popular of the London Charitable Eye Institutions, namely, the Royal Infirmary for the Diseases of the Eye, in Cork-street. It is true that Sir H. Halford, and some other gentlemen are connected, as consultants, with the infirmary; but their appointment is merely nominal, and the whole business is managed by Mr. Alexander, without assistant or clerk. The name "Royal Infirmary" signifies, as with other of the London institutions, nothing farther than that the office-bearers have chosen the king as patron. The infirmary it entirely poli-clinical, and comprised in a very confined set of rooms. Mr. Alexander gives gratuitous advice thrice a week, to from three hundred to four hundred patients. This occupies him two or three hours.

Wondrous is the activity with which in this proportionally short time, Mr. Alexander examines so great a number of patients, determines the diagnosis of their diseases, single-handed enters them in the journal, prescribes for them, dispenses, himself, most of the internal medicines, performs operations of more or less importance on the eye, and maintains, amidst such a crowd, the necessary degree of police. To solve this difficult, comprehensive, and complicated problem, it is so arranged, that the small consultation-room, which is lighted by a sky-light, is connected with the waiting-room by two doors, through one of which the patients enter, while through the other they retire. In the consultation-room is a very convenient arm-chair, the back of which presents a soft hollow space for the reception of the patient's head. In this chair the patient immediately places himself on his entrance, (or the nurse does, if the patient be a child;) and as quickly must he vacate his seat, when he is dispatched about his business, and removes himself through the door of exit. In the consultation-room stand several barrels full of fluid medicines, eye-waters of different sorts. From these Mr.

Alexander taps, as he speaks to the patient, and measures, by his eye, the necessary quantities into the bottle which the patient brings with him, at the same time putting into the patient's hand a printed paper of directions. These directions are occasionally full and particular. Those for ophthalmia neonatorum appeared very proper, and were well put together.

Except this ophthalmia, Mr. Alexander regards all the other inflammations of the eye in children as scrofulous. In adults, he appears to know only three ophthalmiæ, viz. iritis, xerophthalmia, and psorophthalmia. Under this last are comprehended, in London, almost all inflammations of the eyelids, with slight affections of the eye-ball, catarrhal rheumatic inflammations of a serous, mucous, puriform kind, with or without granulations and growths on the lining membrane of the lids. Xerophthalmia, again, comprehends those more deeply-seated affections of the eyeball, not perhaps distinctly inflammatory, often more of a congestive nature, and sometimes subamaurotic. The diagnosis of the ophthalmiæ in England extends no further than the distinguishing of these few varieties.

Xerophthalmia, as it is called, arises chiefly from long-continued straining of the eyes by candlelight. It is treated with local bleedings, purges, cooling lotions, and opiates taken at bed-time. In iritis, calomel, and cupping on the temples, are prescribed. In psorophthalmia and strumous inflammations of the eyes, especially if there be fulness of the vessels, with slight swelling of the lining membrane of the lids, Mr. Alexander scarifies the conjunctiva with pretty long incisions. Mr. Alexander introduces red precipitate salve with a spatula between the eyeball and the upper eyelid, at the outer angle. Vinum opii he pours upon the eye with a little spoon; he employs alum-water abundantly; and uses as an escharotic the solid sulphate of copper.

Mr. Alexander says that he has cured blenorrhœa of the lacrymal sac by means of frequent, long-continued pressure of the contents of the sac through the nasal canal.

The operation for cataract, which he generally prefers, is extraction, and his procedure has several peculiarities. The patient sits in the arm-chair already noticed, the head being bent considerably back, and pressed against the hollow space forming the top of the chair-back. This the operator manages himself, while standing behind the patient. With the thumb and forefinger of the hand which is disengaged, he fixes the edges of the upper and under eyelids towards the nose, pressing them against the margins of the orbit, and thus keeping the eyelids open. This was accomplished, as it seemed to us, with considerable security. The section of the cornea was made with Wenzel's knife, in the direction upwards and somewhat outwards. In this part of the business, the operator went somewhat slowly to work, tarrying long with the knife in the anterior chamber, pressing the instrument on with pauses, during which he addressed himself to the patient, exhorting him to quietness, and receiving from him pretty full replies. To divide the capsule, Mr. A. makes use of a hook with a sharp point, made of gold, and contrived by Phipps. He enters this hook with great steadiness, pushing it through the pupil into the posterior chamber, driving its point far behind the iris towards the nasal angle of the eye, drawing it across towards the temple, and so effecting a horizontal rent of the capsule. With moderate pressure on the eyeball, the lens escaped whole and entire. We saw Mr. A. perform several extractions in this way with complete success. The cases, indeed, were of the most favorable sort; pure, hard, lenticular cataracts, of moderate size, without any opacity of the capsule, in old, very composed subjects, with large anterior chambers, and ordinary prominence of the eyeballs. After all, however, the technical skill of Mr. A. is very great, and he must unquestionably be ranked amongst the best of operators. Whether he be one of the best and most intelligent oculists is another question. The operation being finished, he lays a wet linen compress over the closed eye, and fastens this with a roller.

Whether there be any one in London besides Mr. Alexander possessing a real taste for eye-operations, we might almost doubt. We saw performed by other hands only one very successful operation on the eye. Mr. Tyrrell formed an artificial pupil by incision of the iris. The case was very favorable for the operation. The iris, much on the stretch, was adherent to the lower part of the cornea, which, except at the place of the adhesion, was sound and completely transparent. The anterior chamber was at the same time sufficiently large; the posterior entire, and without exudation, and the lens and capsule natural. Having made an incision, rather too large, through the cornea, the operator passed a pair of small thin-bladed scissors, bent into an obtuse angle in the direction of their cutting edges, and having one point sharp, and the other blunt, into the anterior chamber. The restlessness of the patient required time and patience. The English operators appear in general, however, to lay but little stress on the quick termination even of eye-operations. At length the horizontal incision through the middle of the iris was completed, when instantly the edges sprang aside, and the pupil gaped widely, especially at its centre. The parts in the posterior chamber did not appear to be disturbed, much less injured.

This closes the list of fortunate eye-operations which we saw in London. All the others failed in the utmost degree. The last-mentioned operator performed, in the Moorfields Ophthalmic Infirmary, a depression through the sclerotica, which miscarried entirely, and in which the eye was materially injured. His colleague attempted an extraction. The section of the cornea happened to be uncommonly small. After he had opened the capsule, he became convinced, only by the fruitlessness of long-continued pressure on the eyeball, of the necessity of enlarging the section. For this purpose there is in use in London, not Dariel's scissors, but a narrow little probe-pointed knife, with a concave cutting edge, introduced by Phipps. This stupid instrument was six times introduced into the anterior chamber, and the collapsed cornea, incapable of offering the necessary resistance to be divided with a knife, but drawn by the instrument into folds, was at length sawn, rather than cut.

Still more unfortunate was the result of an extraction undertaken by Mr. Earle. In this case, too, the section of the cornea was too small, and quite irregular, and the iris besides was wounded. Dilatation of the section with the probe-pointed knife was had recourse to, then attempts made by pressure to extract the lens, then repeated introductions of the curette for the same purpose, till at length, after fruitless endeavours for half an hour, the lens was left in the eye, and the unfinished operation delared impracticable.

The Moorfields Eye Infirmary, of which mention has just been made, is, in other respects, well situated, and fitly conducted. Besides lodging for the superintendent and house-surgeon, it contains a spacious consultation-room, where, amidst a throng of pupils, the poli-clinic is conducted, not in a way, I think, likely to be very instructive,—an operation-room and several wards, each containing ten beds, where lie the patients that have undergone operations. Lawrence was formerly, Tyrrell is now, the chief medical attendant on this institution.

Besides these, there is a third Eye Infirmary, the Royal Westminster, under the direction of Mr. Guthrie. This hero of English military surgery also treats eye-cases with heroic means. We saw in his institution a well-marked case of syphilitic iritis, the nature of which he had not discerned, but had treated, according to his well-known method, with the internal exhibition of oil of turpentine. In blenorrhœal and granulating inflammations of the eye, he puts in upon the eye, with a wooden spatula, large portions of a salve, made up of six grains of Lapis Infernalis, ten drops of Goulard, and half an ounce of Axunge; and rubs it in very much with the eyelids. He calls this "Unguentum ophthalmicum magicum," having seen quite extraordinary and almost incredible effects from its use. The pain which it causes is very great and continued. The traumatic reaction goes off only after several days. According to his notion, a peculiar Egyptian ophthalmia has at no time been prevalent among the English troops, and those who assert the contrary he accuses of quackery and fraud. On this point he expresses himself violently respecting Adams, against whom indeed the surgeons, generally, of London, are extremely bitter. Adams, after receiving from Parliament a national remuneration for his pretended discovery of granulations as a characteristic and diagnostic sign of the Egyptian ophthalmia, and living in style as a rich man, lost his means by unfortunate speculations in the stocks, and has disappeared from London, without our being able to learn if he were yet alive, or whither he had gone. We could not join in the unconditional reprehension and contemptuous verdict of his opponent. Supposing even that his accomplishments as an author were of no great worth, and his numerous works more calculated to commend his own operations and to secure patients, than to advance the interests of knowledge, still would he claim a lasting merit and enduring fame, had he written nothing more than his small publication on Ectropium, and on the operation which he practised for the cure of that disease, which was really a useful, praiseworthy, and real advancement of the art, in a department which in a great measure had previously been studied to no purpose.—[*Translated from the German for The Lancet, by* ALLAN GREAME, M.D.]

### ANECDOTE OF THE DOMESTIC CAT.

The sagacity of animals in shunning disease has been observed by several naturalists, particularly among gregarious animals, who are constantly found to avoid such as are affected with any complaint. Domestic animals are not generally so remarkable for this propensity, as their habits are associated with those of man. A cat I have, however, has exhibited a remarkable instance of sagacity during the present epidemic. She had been in the practice of coming up to my bed-room every morning to drink out of my ewer; but, during the continuance of the febrile symptoms attending the influenza, she did not come to drink, and never entered the room. No sooner, however, had the fever subsided, than she immediately returned, and took her morning draught as usual. This shows that the senses of animals, particularly that of smell, must be extremely acute, and that the diseased atmosphere, however insensible we may be to its effect, has a powerful influence on their more acute organs. —*Field Naturalist's Magazine.*

### DIFFICULTIES OVERCOME BY SCIENCE.

The manner in which facts apparently lost are restored to light, even after considerable intervals of time, is sometimes very unexpected; and a few examples may not be without their use. The thermometers employed by the philosophers, who composed the Academia del Cimento, have been lost; and, as they did not use the two fixed points of freezing and boiling water, the results of a great mass of observations have remained useless, from our ignorance of the value of a degree on their instrument. M. Libri, of Florence, proposed to regain this knowledge by comparing their registers of the temperature of the human body and of that of some warm springs in Tuscany, which have preserved their heat uniform during a century, as well as of other things similarly circumstanced.

Another illustration was pointed out to me by M. Gazzeri, the professor of Chemistry, at Florence. A few years ago, an important suit in one of the legal courts of Tuscany depended on ascertaining whether a certain word had been erased by some chemical process from a deed then before the court. The party who insisted that an erasure had been made availed themselves of the knowledge of M. Gazzeri, who, concluding that those who committed the fraud would be satisfied by the disappearance of the colouring matter of the ink, suspected (either from some colourless matter remaining in the letters, or perhaps from the agency of the solvent having weakened the fabric of the paper itself beneath the supposed letters,) that the effect of the slow application of heat would be to render some difference of texture or of applied substance evident, by some variety in the shade of colour which heat in such circumstances might be expected to produce. Permission having been given to try the experiment, on the application of heat the important word reappeared, to the great satisfaction of the court.—*Babbage's Reflections on the Decline of Science in England.*

### COST OF BECOMING A MAN OF LETTERS.

It is the custom to attach certain letters to the names of those who belong to different societies, and these marks of ownership are by many considered the only valuable part of their purchase on entry. The following is a list of some of these societies. The second column gives the ready-money prices of the tail-pieces indicated in the third.

| SOCIETIES. | Fees on Admission, including Composition for Annual Payments. £ s. d. | Appended Letters. |
|---|---|---|
| Royal Society .................... | 50 0 0 | F.R.S. |
| Royal Society of Edinburgh... | 25 4 0* | F.R.S.E. |
| Royal Academy of Dublin ... | 26 5 0 | M.R.I.A. |
| Royal Society of Literature... | 36 15 0 | F.R.S. LIT. |
| Antiquarian ..................... | 50 8 0 | F.A.S. |
| Linnæan........................... | 36 0 0 | F.L.S. |
| Geological ........................ | 34 13 0 | F.G.S. |
| Astronomical .................... | 25 4 0 | M.A.S. |
| Zoological ........................ | 26 5 0 | F.Z.S. |
| Royal Institution ............... | 50 0 0 | M.R.I. |
| Royal Asiatic ................... | 31 10 0 | F.R.A.S. |
| Horticultural..... ............... | 48 6 0 | F.H.S. |
| Medico-Botanical .............. | 21 0 0 | F.M.B.S. |

Thus, those who are ambitious of scientific distinction, may, according to their fancy, render their name a kind of comet, carrying with it a tail of upwards of forty letters, at the average cost of 10*l.* 9*s.* 9¼*d.* per letter.—*Ibid.*

### DR. THOMAS YOUNG.

Now I am persuaded that there does not exist at this day in the profession an individual who comes up to the standard which (it is implied) all ought to reach. It has been my happiness to know many men in my time who have had enough of attainment to command my highest respect; some who have reached great eminence during their lives, and some who have been thought worthy of mo-

* The Royal Society of Edinburgh now requires, for composition in lieu of annual contributions, a sum dependent on the value of the life of the member.

numents since their deaths; yet I have known one, and one only, who came up to the requirements of an introductory lecture, which I have read; and that was Dr. Thomas Young. But Dr. Young stood alone among mankind. The most learned and scientific men of his time were struck with wonder at the extent and variety of his knowledge: yet Dr. Young was the only person whom any man now alive ever saw learned and scientific enough for a physician, according to the Utopian measure of things.—*Dr. Latham, in the Med. Gazette.*

### HOTTENTOT'S BREAD.

The *Testudinaria elephantipes*, or Hottentot's bread, is a very curious plant, resembling in its rootstock a tortoise encased in its protective shell. My friend, Mr. Burchell, to whom we are so much indebted for information collected during his travels in Africa, tells me he met with it frequently; and in times of scarcity the Hottentots break off the woody case, and eat the pithy substance it contains, whence the name Hottentot's bread.—*Burnett's Outl. of Botany.*

*Testudinaria elephantipes.* *(a)* Flower. *(b)* Rootstock, voluble stem, and reticulate leaf.

### ARBOREOUS FERNS.

The ferns of this and other temperate regions are comparatively humble plants, their true stems generally creeping on the surface of the earth, as in the *Lycopodiaceæ*, or even being subterranean, as in most of the frondose ferns, the parts which are usually considered stems being in reality only branches; but in the West Indies, in St. Helena, the Isle of Bourbon, and other hot insular situations, arboreous species are found, the stems of which rise out of the earth, and elevate their crown of fronds to the height of twenty, thirty, or forty feet, or even more. In the British Museum is a stem of *Alsophila*, brought to England by Dr. Wallich, that measures forty-five feet; but seventy or eighty feet are occasionally attained. In hese noble examples of the class, the true structure of the stem and affinities of the plants in general with palms, and even with cycases and pines, is much more obvious, even to the common observer, than in the suffruticose and herbaceous brakes that are now indigenous to these northern latitudes. Even the Aspidia or shield-ferns, which do form a dwarfish stem and collect their fronds into a crown, hold only the same comparative rank to the arboreous species as onions or lilies do to palms; or our herbaceous cresses to our forest-trees; and the ferns with subterranean stems, like our eagle-brakes and horsetails, are only to be compared with the tree-ferns in the same way in which fodder-grasses are compared to canes and towering bamboos, or rushes to the loftiest palms.—*Ibid.*

*Cyathea glauca,* and other arboreous ferns, natives of the Brazils and of the Isle de Bourbon.

### EASY PARTURITION AMONG SAVAGES.

In confirmation of these accounts, which are not always received by the fireside philosopher with the credit which they frequently deserve, I may be allowed to mention an anecdote related to me by a very intelligent American, who four or five years ago was a pupil of this class, and is now prosecuting the practice of physic in his own country. This gentleman, in October 1822, was sailing down the American river St. Lawrence, in company with a party of natives: among them was a pregnant female, who complained of being ill, was landed, retired a little distance from the shore, and returned in an hour to the boat, which waited for her, with an infant, that she had just brought into the world. I place every reliance on this statement. I hold in my hand my pupil's original account, written in French, and he assured me that the account was by no means looked upon as extraordinary by the people with whom he journeyed.—*Dr. Ramsbotham's Lectures in Medical Gazette.*

RAPID PARTURITION, AS CONNECTED WITH FORENSIC MEDICINE.

A woman has been known, I might almost say, to drop a child, while moving across the drawing-room, while riding in a coach, while walking in the street, under circumstances in which her own life, as well as the infant's, must be brought into some degree of hazard.

Now the possibility of such an occurrence is worth being borne in mind, not only that we may guard our married patients, if we know they possess a large pelvis, (and we may know it by having attended them in labour previously,) against the chance of the child suddenly emerging into the world, but in a judicial point of view also. It may be the means of saving a life that might have been unjustly sacrificed, if we reflect how rapidly the process of labour has been accomplished in some instances; and that what has once occurred in nature may possibly occur again. We will presume, then, that a young unmarried woman has unfortunately been entangled by the snares of one of our own sex, and that she finds herself impregnated: from the modesty inherent in the female character, she is unwilling that her shame should be divulged; she fully intends to acknowledge her misfortune before her expected accouchement, but puts it off from day to day, with a natural aversion to make a confession which must drive her from the society of her friends. Probably she may have miscalculated the time at which she expects to be delivered, or perhaps she may be seized with parturient pains rather prematurely. She feels a rumbling in her bowels, (by no means uncommon at the commencement of labour;) she fancies she has an inclination to evacuate the rectum; she retires to the garden for that purpose; she finds strong pains come on; she is unable to leave the seat on which she has placed herself; the vagina and the external parts are very much relaxed; the uterus acts forcibly, and the child and placenta are propelled out together into the mire below. She is aware then of what has happened; she hears no scream; she has but a very few minutes in which to take a final determination; she wishes to hide the occurrence; she comes into the house again; returns to her friends; makes the best story she can for the appearance of her linen; and for a time everything is hushed. By-and-by suspicion arises: it is possible the place may be emptied, and the child is found. A cry of "murder!" is raised; she is suspected first, then indicted, dragged into a court of justice, and upon medical testimony will often depend her life.

That a girl inexperienced in the feelings of childbirth may be unaware of the approach of labour, is sufficiently evident to require no illustration; but I cannot help enforcing the position by relating a short anecdote. I was in attendance on a lady who had borne a number of children, and had generally suffered protracted labours. I had just made an examination, and found the os uteri but little dilated. I was standing by the fire in her chamber, in conversation with a female relative, who was anxiously watching over her, when I was requested to leave the room, in consequence of an inclination my patient felt to evacuate the rectum. I had scarcely retired when I was summoned back again; and, to my surprise, I found her sitting on the night-table, and both child and placenta lying in the pan beneath. In this instance the infant was saved. Had the same accident happened in a closet, it would most probably have lost its life. This case shews both the rapidity with which the parts occasionally become dilated, and also how much the mother herself may be deceived in her sensations. Nor is this the only instance of the kind that I have witnessed; but another very similar has also come under my own immediate notice.

It is very possible, then, that such an occurrence may take place as I have attempted to bring before you, and under such circumstances let us always lean to the side of mercy. We must not, and we dare not, as we value our oath, state what we know to be untrue, and we should be equally criminal if we withheld any knowledge that we possessed bearing on the case: we are bound to give in evidence that such rapid labours have occasionally happened, and it is for the judge and jury to grant the poor creature the benefit of this information. Let us treasure up these facts in our recollection; we may hereafter, perhaps, derive much consolation from their application.—*Ibid.*

### THE DISEASES AND CASUALTIES THIS YEAR, FROM DECEMBER 11TH, 1832, TO DECEMBER 10TH, 1833.

*(From the Bills of Mortality.)*

| DISEASES. | |
|---|---|
| Abscess | 131 |
| Age, and Debility | 2952 |
| Apoplexy | 442 |
| Asthma | 1265 |
| Cancer | 105 |
| Childbirth | 275 |
| Cholera | 1150 |
| Consumption | 4355 |
| Constipation of the Bowels | 26 |
| Convulsions | 2140 |
| Croup | 151 |
| Dentition or Teething | 473 |
| Diabetes | 6 |
| Diarrhœa | 19 |
| Dropsy | 860 |
| Dropsy on the Brain | 860 |
| Dropsy on the Chest | 100 |
| Dysentery | 5 |
| Epilepsy | 28 |
| Erysipelas | 82 |
| Fever | 530 |
| Fever, Intermittent or Ague | 13 |
| Fever, (Scarlet) | 481 |
| Fever, (Typhus) | 100 |
| Fistula | 3 |
| Gout | 53 |
| Hæmorrhage | 42 |
| Heart, diseased | 145 |
| Hernia | 29 |
| Hooping Cough | 1040 |
| Hydrophobia | 4 |
| Indigestion | 9 |
| Inflammation | 2607 |
| Inflammation of the Bowels and Stomach | 499 |
| Inflammation of the Brain | 236 |
| Inflammation of the Lungs and Pleura | 548 |
| Influenza | 135 |

| | | | |
|---|---|---|---|
| Insanity | 142 | Thrush | 109 |
| Jaundice | 55 | Tumour | 43 |
| Jaw-locked | 6 | Venereal | 6 |
| Liver, diseased | 302 | Worms | 2 |
| Measles | 524 | Unknown Causes | 887 |
| Miscarriage | 20 | Stillborn | 934 |
| Mortification | 241 | CASUALTIES. | |
| Paralysis | 212 | Drowned | 108 |
| Rheumatism | 37 | Died by Visitation of God | 39 |
| Scrofula | 19 | Excessive Drinking | 5 |
| Small Pox | 574 | Found dead | 8 |
| Sore Throat and Quinsey | 57 | Killed by various Accidents | 169 |
| Spasm | 79 | Murdered | 4 |
| Stone and Gravel | 91 | Poisoned | 6 |
| Stricture | 16 | Suicides | 55 |

Christened { Males, 13553 } Total, { Females, 13537 } 27090 | Buried { Males, 13319 } Total, { Females, 13258 } 26577.

Of the number Buried were, Stillborn, 934; Under Two Years of Age, 6261; Two and under Five Years, 2805; Five and under Ten, 1145; Ten and under Twenty, 970; Twenty and under Thirty, 1700; Thirty and under Forty, 2225; Forty and under Fifty, 2615; Fifty and under Sixty, 2412; Sixty and under Seventy, 2551; Seventy and under Eighty, 2043; Eighty and under Ninety, 802; Ninety and under a Hundred, 107; One Hundred, 3; One Hundred and One, 1; One Hundred and Two, 1; One Hundred and Three, 1; One Hundred and Four, 1.

Decrease in the Burials reported this year 2029.

## Figures and Tables

**Figure f1:**
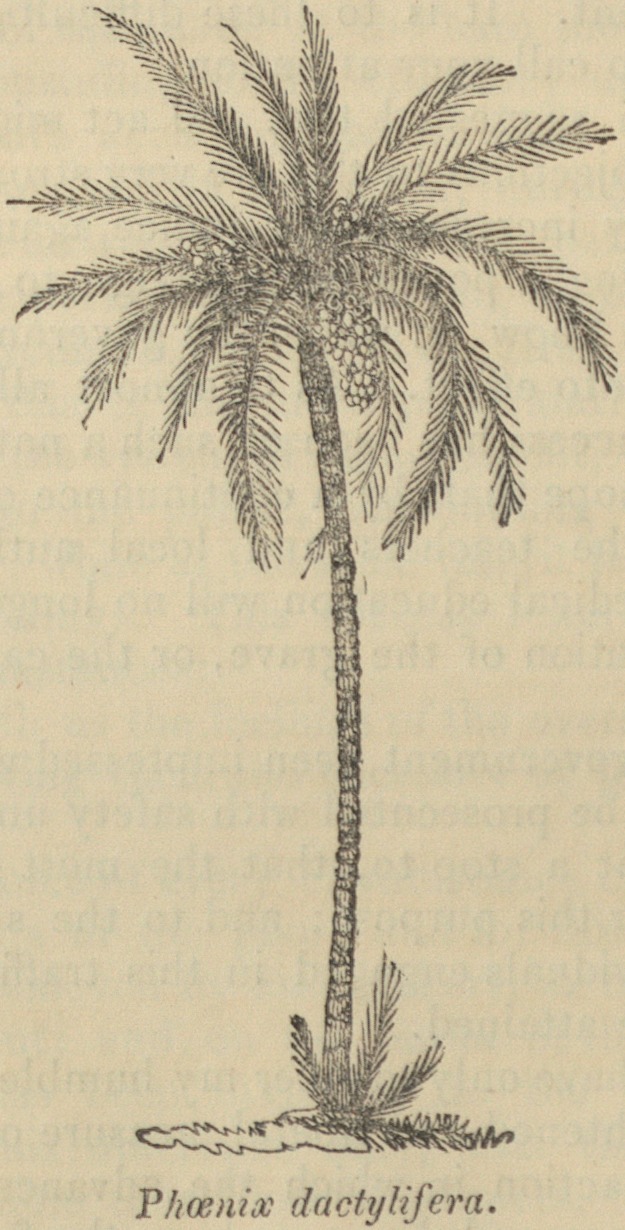


**Figure f2:**
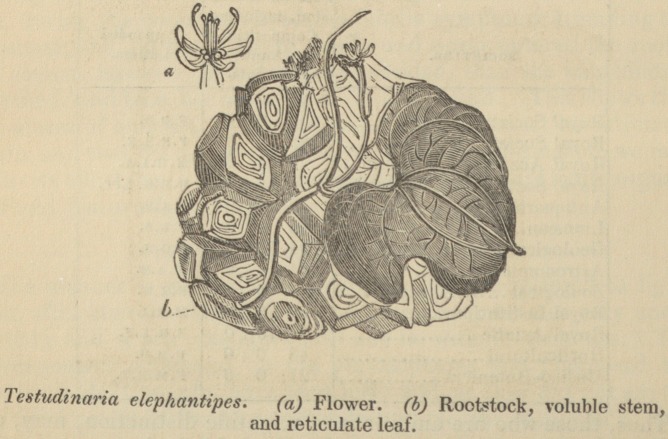


**Figure f3:**